# Índice de Imunoinflamação Sistêmica e Isquemia em Pacientes com Artérias Coronárias Não Obstrutivas

**DOI:** 10.36660/abc.20240081

**Published:** 2024-04-09

**Authors:** Henrique Tria Bianco

**Affiliations:** 1 Universidade Federal de São Paulo São Paulo SP Brasil Universidade Federal de São Paulo (UNIFESP), São Paulo, SP – Brasil

**Keywords:** Doença da Artéria Coronariana, Isquemia Miocárdica, Vasos Coronários, Dor no Peito

A doença cardiovascular é a principal causa de morte em todo o mundo e a doença arterial coronariana (DAC) é o tipo mais comum de doença cardiovascular. No entanto, uma parcela significativa dos pacientes que apresentam desconforto torácico característico não demonstra DAC obstrutiva, definida como estenose ≥50% em pelo menos uma artéria coronária na angiografia. Esses pacientes geralmente recebem diagnósticos de afecções não cardiológicas, como distúrbios gastrointestinais ou de desordens psicossomáticas.^1^ Especula-se que a disfunção vascular coronariana parece ser a causa subjacente da isquemia em grade parte destes casos. Desta forma, a padronização dos critérios diagnósticos para sintomas isquêmicos devido à disfunção microvascular coronariana (DMV) é necessária para investigação adicional de pacientes que apresentam dor torácica anginosa consistente com "angina microvascular".^2^

Historicamente, os únicos métodos práticos disponíveis para avaliação têm sido invasivos, como o doppler intracoronário ou a termodiluição. Isto provavelmente prejudicou a avaliação objetiva da DMV em pacientes que apresentam dor torácica sem DAC obstrutiva. Assim, o tratamento tem sido frequentemente estudado em entidades clínicas imprecisas, como a síndrome cardíaca X.^3 , 4^ Além disso, a falta de consenso sobre critérios diagnósticos e nomenclatura obscureceu ainda mais as evidências que buscavam definir objetivamente a angina microvascular como uma entidade clínica distinta.^5^

No cenário e contexto das síndromes coronárias, há vários marcadores inflamatórios, como a proteína-C reativa (PCR), fator de necrose tumoral-α e várias interleucinas, que estão associados a um desfecho pior.^6^ Embora a inflamação sistêmica grave seja um indicador estabelecido de mortalidade na síndromes coronárias agudas (SCA), nenhum biomarcador inflamatório único é capaz de orientar o tratamento do risco cardiovascular.^7^ Por sua vez, índices hematológicos simples, como a relação neutrófilos-linfócitos (RNL) e a relação plaquetas-linfócitos, também são indicadores úteis e promissores para a estratificação acurada da doença cardiovascular.^8^ Nas SCA, a ativação descontrolada da imunidade inata e adaptativa converge com a ativação plaquetária, resultando na formação de trombos. O índice imunoinflamatório sistêmico (IIS), derivado das contagens de plaquetas, neutrófilos e linfócitos, combina os principais atores dessas vias fisiopatológicas, sendo descrito pela primeira vez como uma ferramenta prognóstica no carcinoma hepatocelular.^9^

O IIS foi relacionado com a extensão do dano miocárdico e essa relação provavelmente se deu por meio do tipo de SCA, com valores mais elevados deste índice em pacientes com infarto com Supra-ST (IAMCSST). Este achado está de acordo com estudos anteriores, cujos resultados demonstraram que, na ausência de necrose, a correlação da contagem de leucócitos com a mortalidade diminuiu.^10 , 11^

O manuscrito intitulado "A Relação entre o Índice de Imuno-Inflamação Sistêmica e Isquemia com Artérias Coronárias Não Obstrutivas em Pacientes Submetidos à Angiografia Coronária" investigou a relação entre isquemia em artérias coronárias não obstrutivas (INOCA) e o índice de imunoinflamação sistêmica (IIS) que trata da relação plaquetas × neutrófilos/linfócitos.^12^

Um total de 424 pacientes com idade média de 56 anos foram incluídos. Estes foram alocados em dois grupos de acordo com o diagnóstico de INOCA. Como resultado, observou-se que os pacientes com INOCA eram mais propensos a uma contagem mais elevada de plaquetas, relação neutrófilos para linfócitos, e valores de IIS. O valor de corte ideal do IIS para prever o INOCA foi 153,8, com sensibilidade de 44,8% e especificidade de 78,77%. O valor da AUC do IIS foi maior do que o dos linfócitos e plaquetas em pacientes INOCA.

Desta forma, a despeito de algumas limitações do estudo, os autores concluem que um IIS elevado pode estar associado ao aumento da atividade inflamatória. Desta forma, esse achado sugere que o IIS pode potencialmente ser usado para identificar e estratificar indivíduos de mais alto risco, ainda na admissão em unidades hospitalares.
