# Supplementary material for: Índice de Imunoinflamação Sistêmica e Isquemia em Pacientes com Artérias Coronárias Não Obstrutivas
Source: Arq Bras Cardiol. 2024 Apr 9;121(2):e20240081. [Article in Portuguese] doi: 10.36660/abc.20240081 (PMC11081205; doi:10.36660/abc.20240081)
Supplement: Translation

# Translation: Index of Systemic Immunoinflammation and Ischemia in Patients with Non-Obstructive Coronary Arteries

**Authors:** Henrique Tria Bianco

**Affiliations:** 1 São Paulo SP Brazil Universidade Federal de São Paulo (UNIFESP), São Paulo, SP – Brazil

**Keywords:** Coronary Artery Disease, Myocardial Ischemia, Coronary Vessels, Chest Pain

Cardiovascular disease is the leading cause of death worldwide and coronary artery disease (CAD) is the most common type of cardiovascular disease. However, a significant portion of patients who present with characteristic chest discomfort do not demonstrate obstructive CAD, defined as ≥50% stenosis in at least one coronary artery on angiography. These patients are generally diagnosed with non-cardiological conditions, such as gastrointestinal disorders or psychosomatic disorders.^1^It is speculated that coronary vascular dysfunction appears to be the underlying cause of ischemia in a large proportion of these cases. Therefore, standardization of diagnostic criteria for ischemic symptoms due to coronary microvascular dysfunction (CMD) is necessary for further investigation of patients presenting with anginal chest pain consistent with "microvascular angina".^2^

Historically, the only practical methods available for assessment have been invasive, such as intracoronary Doppler or thermodilution. This probably hindered the objective assessment of CMD in patients presenting with chest pain without obstructive CAD. Thus, treatment has often been studied in imprecise clinical entities such as cardiac syndrome distinct.^3 , 4^Furthermore, the lack of consensus on diagnostic criteria and nomenclature has further obscured the evidence that sought to objectively define microvascular angina as a distinct clinical entity.^5^

In the setting and context of coronary syndromes, there are several inflammatory markers, such as the C-reactive protein (CRP), tumor necrosis factor-α, and several interleukins, which are associated with a worse outcome.^6^Although severe systemic inflammation is an established indicator of mortality in acute coronary syndromes (ACS), no single inflammatory biomarker is capable of guiding the treatment of cardiovascular risk.^7^In turn, simple hematological indices, such as the neutrophil-lymphocyte ratio (NLR) and the platelet-lymphocyte ratio, are also useful and promising indicators for the accurate stratification of cardiovascular disease.^8^In ACS, uncontrolled activation of innate and adaptive immunity converges with platelet activation, resulting in thrombus formation. The systemic immunoinflammatory index (IIS), derived from platelet, neutrophil, and lymphocyte counts, combines the main actors of these pathophysiological pathways, being described for the first time as a prognostic tool in hepatocellular carcinoma.^9^

The IIS was related to the extent of myocardial damage and this relationship was probably due to the type of ACS, with higher values of this index in patients with STEMI infarction. This finding is in line with previous studies, whose results demonstrated that, in the absence of necrosis, the correlation between leukocyte count and mortality decreased.^10 , 11^

The manuscript titled " *The Relationship between the Systemic Immune-Inflammation Index and Ischemia with Non-Obstructive Coronary Arteries in Patients Undergoing Coronary Angiography* " investigated the relationship between ischemia in non-obstructive coronary arteries (INOCA) and the systemic immunoinflammation index (IIS), which deals with the platelet × neutrophil/lymphocyte ratio.^12^

A total of 424 patients with a mean age of 56 years were included. These were allocated into two groups according to the INOCA diagnosis. As a result, it was observed that patients with INOCA were more prone to a higher platelet count, neutrophil-to-lymphocyte ratio, and IIS values. The ideal IIS cutoff value to predict INOCA was 153.8, with a sensitivity of 44.8% and a specificity of 78.77%. The AUC value of IIS was higher than that of lymphocytes and platelets in INOCA patients.

Therefore, despite some limitations of the study, the authors conclude that a high IIS may be associated with increased inflammatory activity. Therefore, this finding suggests that the IIS can potentially be used to identify and stratify higher-risk individuals, even upon admission to hospital units.
